# Analysis of Respiratory Sounds: State of the Art

**DOI:** 10.4137/ccrpm.s530

**Published:** 2008-05-16

**Authors:** Sandra Reichert, Raymond Gass, Christian Brandt, Emmanuel Andrès

**Affiliations:** 1 Ph.D., e-health UTBM student, Alcatel-Lucent, Chief Technical Office, Strasbourg, France; 2 Technical Academy Fellow, Alcatel-Lucent, Chief Technical Office, Strasbourg, France; 3 M.D., Head of the Cardiology Department, *Clinique Médicale B, CHRU Strasbourg*, Strasbourg, France; 4 M.D., Ph.D., Head of the Internal Medicine Department, *Clinique Médicale B, CHRU Strasbourg*, Strasbourg, France

**Keywords:** state of the art, auscultation, respiratory sounds, crackles, wheezes, respiratory phase detection, spectral analysis, wavelet, respiratory phase classification, signal processing, artificial neural networks, genetic algorithm, multilayer perceptron, fuzzy rule base identification system

## Abstract

**Objective:**

This paper describes state of the art, scientific publications and ongoing research related to the methods of analysis of respiratory sounds.

**Methods and material:**

Review of the current medical and technological literature using *Pubmed* and personal experience.

**Results:**

The study includes a description of the various techniques that are being used to collect auscultation sounds, a physical description of known pathologic sounds for which automatic detection tools were developed. Modern tools are based on artificial intelligence and on technics such as artificial neural networks, fuzzy systems, and genetic algorithms…

**Conclusion:**

The next step will consist in finding new markers so as to increase the efficiency of decision aid algorithms and tools.

## Introduction

Distinction between normal respiratory sounds and abnormal ones (such as crackles, wheezes…) is important for an accurate medical diagnosis. Respiratory sounds include invaluable information concerning the physiologies and pathologies of lungs and airways obstruction. Thus, the spectral density and amplitude of sounds can indicate the state of the lungs parenchyma, the dimension of the airways and their pathological modification [1].

## Limits of human audition

Studies were performed in order to test the human’s ear capability to detect crackles in an auscultation signal [2]. The methods used consist in simulated crackles superimposed on real breath sound. The results indicate that the most important detection errors are due to the following factors:

- Intensity of the respiratory signal: deep breaths mask more crackles than superficial breaths,
- Type of crackles: fine crackles are easily recognizable in so far as their waveform differs more from the waveform of classical lung sounds,
- Amplitude of crackles.

It can be inferred from these studies that the validation of automatic crackles detection algorithms should not take auscultation as unique reference.

On the contrary, the understanding of mechanisms linked to the creation of breath sounds is, for the moment, imperfect. The recording and analysis of respiratory sounds allow to improve this understanding [3] and an objective relationship between abnormal respiratory sounds with respiratory pathology. Besides, an objective analysis allows to develop classification systems [4] that make it possible to precisely qualify normal and adventitious respiratory sounds.

Whilst conventional stethoscope auscultation is subjective and hardly sharable, these systems should provide an objective and early diagnostic help, with a better sensitivity and reproducibility of the results.

Moreover, applications, including diagnosis establishment, monitoring and data exchange through Internet are obviously complementary tools to objective and automatic auscultation sounds analysis. Sensors devices will allow long duration monitoring for patient at home or at hospital. It could also be a useful solution for less-developed countries and remote communities [5]. In addition, this type of system has the great advantage to keep the non-invasive and less expensive characteristics of auscultation.

Finally, Sestini and coll.’s studies [6] indicate that an association between acoustical signal and its image is beneficial to the learning and understanding for students in medical science.

## Propagation of respiratory sounds

The propagation and deformation of breath sounds are linked to several factors [7]:

- The acoustical response of the stethoscope, the asymmetry of the sounds (that can indicate the presence of a pathology), the heterogeneous composition of the body surface (bones, muscles, skin…) that behave like filters;
- The analysis point: measurements indicate that lung sounds are lower in amplitude than tracheal sounds.

## Definition of common markers

Nowadays, there are several definitions for the typical markers of wheezes and crackles [8]. Thus, a universal semantic has to be created. Several works [9] have attempted to collect definitions of terms relating to respiratory sounds and have arrived at a collection of 162 terms commonly used in the « *Computer Respiratory Sound Analysis* » (CORSA).

Nevertheless, it still doesn’t allow physician to have a common definition of terms that are used. For example, a wheeze is still currently associated to a “whistling sound”, and a crackle to “a sound of rice in a frying pan”.

## Definition of semiology

The article of Rossi and coll. [10] gives recommendations concerning the experimental conditions required for recording respiratory sounds. It describes the optimal experimental conditions (principally concerning background noise, including sounds other than respiratory such as vocal sounds) and the specific procedures according to the type of sounds he wanted to record (breath, cough, snores), information for the recording (diagnosis, evaluation of a therapy, monitoring), the age of subject (baby, infant, child, adult), and the recording method (free field, endobronchial microphone).

Concerning the tests of the lungs functions for the subject preparation; the author leans on the same recommendations than the ERS (*European Respiratory Society*).

Lastly, for short recordings, a sitting position is recommended, but a lay position is preferably for long recordings.

## Definition of Terms

Sovijarvi and coll.’s article [9], published in the *European Respiratory Journal*, provides accurate definitions of currently used terms in pulmonary auscultation domain and sound analysis; the more pertinent are recalled here:

### Sounds

***Adventitious sound***: it relates to additional respiratory sounds superimposed on normal breath sounds. It can be continuous (like wheezes) or discontinuous (such as crackles). Some of them (like squawks) have both characteristics. The presence of such sounds usually indicates pulmonary disorders.

***Breath sound***: it includes normal and adventitious sounds recorded over the chest wall, the trachea or at the mouth. Their generation is related to airflow in the respiratory tract. Acoustically, this sound is characterized by broad-spectrum noise with a frequency range depending on the pick-up location.

***Lung sound***: it concerns all respiratory sounds heard or detected over the chest wall or within the chest including breath sounds and adventitious sounds detected at this location.

***Normal breath sound***: on the chest wall, respiratory sound is characterised by a low noise during inspiration, and hardly audible during expiration. On trachea, normal respiratory sound is characterized by a broader spectrum of noise (for example containing higher-frequency components), audible both during inspiratory and expiratory phase.

### Known trackers

***Crackles***: these adventitious explosive and discontinuous sounds appear generally during inspiratory phase. They are characterised by their specific waveform, their duration, and their location in the respiratory cycle. A crackle can be characterized by its total duration, as fine (short duration) or coarse (long duration). Occurrences of crackles in lung sounds usually reflect a pathological process in pulmonary tissue or airways.

***Cough sound***: transient sound induced by the cough reflex with a frequency content between 50 and 3000 Hz. The characteristics of cough sounds are different in several pulmonary diseases. Cough sounds containing wheezes are typical in asthma.

***Rhonchus***: rhonchus is a low-pitched wheeze containing rapidly damping periodic waveforms with a duration of >100 ms and frequency of <300 Hz. Rhonchus can be found, for example, in patients with secretions or narrowing in large airways and with abnormal airway collapsibility.

***Snoring sound***: it is a respiratory low-frequency noisy sound with periodic components (fundamental frequency 30–250 Hz) detected usually during sleep induced by abnormal vibrations in the walls of the oropharynx. It is typical inspiratory sound but a small expiratory component can appear especially in patients with obstructive sleep apnea.

***Squawk***: with relatively short inspiratoy adventitious sound having a musical characteristic, occasionally found in patients with interstitial lung disorders. Acoustically, its waveform may resemble that of short wheezes, but they are often preceded by a crackle. The duration of squawks may vary between 50 and 400 ms. The basic mechanisms of their origin probably differ from those of wheezes in obstructive lung diseases.

***Stridor***: it is a very low-frequency wheeze originating in the larynx or trachea. It appears most frequently during inspiration. It can be audible at the mouth, at the trachea and over the chest wall. Stridor can appear, for example, in whooping cough, and in laryngeal or tracheal stenosis.

***Wheeze***: this adventitious and continuous sound presents a musical character. Acoustically, it is characterized by periodic waveforms with a dominant frequency usually over 100 Hz and with duration of ≥100 ms; hence, the sound must include at least 10 successive vibrations. Wheezes are usually associated with airways obstruction due to various causes. If the wheeze contains essentially a single frequency, the wheeze is called monophonic. If it contains several frequencies, it is termed a polyphonic wheeze.

### Visualisation methods

**Figure 1.**
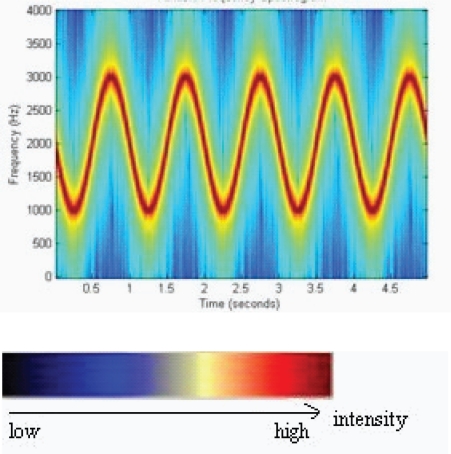
Example of Spectrogram.

***Phonopneumogram***: it is a simultaneous and overlapped display of sound signal and airflow in time domain during breathing:

***Spectrogram***: it concerns representation in which time is represented in abscises, frequency in ordinate, and the intensity of the signal by a palette of colors (Fig. 1).

### Analysis methods

***Artificial neural network*** (ANN): it is a mathematical model based on biological neural networks. It consists in an interconnected group of artificial neurons and processes information using a connectionist approach to computation. Generally, it is an adaptive system that changes its structure based on external or internal information that flows through the network during the learning phase. A ***perceptron*** is a specific type of artificial neural network, that can be seen as the simplest kind of feedforward neural network: a linear classifier.

***k-nearest neighbor algorithm*** (kNN): it is a method for classifying objects based on closest training examples in the feature space. *k*-NN is a type of instance-based learning, or lazy learning where the function is only approximated locally and all computation is deferred until classification.

***Genetic algorithm***: it is a search technique used to find exact or approximate solutions to optimization and search problems. Genetic algorithms are categorized as global search heuristics. They are a particular class of evolutionary algorithms that use techniques inspired by evolutionary biology such as inheritance, mutation, selection, and crossover.

***Fuzzy logic***: it is derived from fuzzy set theory dealing with reasoning that is approximate rather than precisely deduced from classical predicate logic. It can be thought of as the application side of fuzzy set theory dealing with well thought out real world expert values for a complex problem.

***Wavelet:*** it is a kind of mathematical function used to divide a given function into different frequency components and study each component with a resolution that matches its scale. Wavelet transforms have advantages over traditional Fourier transforms for representing functions that have discontinuities and sharp peaks, and for accurately deconstructing and reconstructing finite, non-periodic and/or non-stationary signals.

## Capture Techniques

An adapted capture chain of the sound is a relevant point preceding the analysis phase [11][12][13]. Typically, it is made up of the following elements [3]:

- Sound capturing: the positioning of the microphone is important; actually the chest acts like a reducer and a low-pass filter. Kraman and coll. [14] studied the effects of different microphones and conclude that the most adapted was the electret microphone with conical coupler and a diameter from 10 to 15 mm;
- Amplification of the signal;
- Filtering and sampling;
- Reduction of the cardiac sound;
- Sound recording.

Cheetham and coll.’s article [15] develops the important points related to the digitalisation of the auscultation sounds’ records; it deals with sampling frequency, filtering, signal noise rapport that is introduced by the analogue/digital conversion.

### Acquisition

Various methods and tools have been described to capture sound:

- *Using a unique microphone:* It is the more frequently used method. The sensor is generally an electret microphone, the sampling frequency the most frequently used is the same as the one used for telephony codecs (8kHz), an analogue/digital conversion with a 16bits resolution [16]. Others make use of an accelerometer; it is less sensitive to background noise [17], but performance is must less than an electret microphone.
- *Utilisation of several microphones and three dimensional representations*. This technique makes it possible to identify the location of the origin of the sounds; it is a dynamic method at shows structural and functional properties for diagnosis [18][19].
- *Emission of a sound and analysis of its propagation*. This technique, described in [20], consists in emitting a sound with a loudspeaker introduced in the patient’s mouth. The method processed the characteristics of signal’s propagation through respiratory airways and chest. The analysed parameters are energy ratios, signal time delays, and dominant frequency.
- *Measurement in closed loop controlled ventilation* [21][22].

In our study, we will focus on the use of a unique microphone.

### Filtering and heart sound cancelling

Heart sounds can introduce perturbations during the analysis of lung sounds. Most of the spectrum of heart sounds is located between 20 and 100 Hz. According to Elphick and coll.’s article [23], the attenuation of heart sounds is obtained thanks to a simple band-pass filter [50 Hz, 2500 Hz]. Nevertheless, a high-pass filter at 100Hz is not a good solution in so far as the main components of lung sounds are also located in this frequency range. Consequently, several methods have be tested [24]: wavelets, adaptative filtering with recursive least squares algorithm, time/frequency filtering, reconstruction, AR/MA estimation (autoregressive/mobile average) in time/frequency domain of wavelet coefficients, independent component analysis, and entropy based method.

The filter proposed by Bahoura and coll. [25]is based on a wavelet packet transform, and the use of two filters which are defined in frequency and time domain. This filter provides more accurate and effective results than its rivals; experimental tests demonstrate very good performances. Moreover, the proposed technique allows better care of the characteristics of stationary signals (normal sounds or wheezes).

Yadollahi and coll. [26] try to detect the segments of sound including heart sound, in order to suppress the heart components. They investigate methods using Shannon’s entropy, Renyi’s entropy and multiresolution product of wavelet coefficients. The most efficient method was Shannon’s entropy.

Among all these methods, the better results were obtained with adaptive filtering [27], time/frequency filtering and AR/MA estimation.

### Deleting interference noises

The “cleaning” of respiratory sounds must also take care of the reduction of background sound. This processing can be realized through two different methods [3]: noise reduction through adaptative filtering (deleting white Gaussian noise, deleting vocal sound, reducing measurement errors), and noise reduction through wavelet packets (Donoho’s method…). The more recent techniques use simultaneous usage of several sensors.

## Lung Sounds Characteristics

It is commonly admitted that lung sounds’ frequency is in the frequency range [50, 2500 Hz], and that tracheal sounds can reach up to 4000 Hz; this allows to define a sampling frequency at 8 kHz. The spectrum of heart sounds is defined between 20 and 100 Hz for basic signals and higher frequency (upper than 500 Hz) for breaths.

Abnormal sounds can be divided into two sub-classes [25]:

- Continuous or stationary sounds, like wheezes, rhonchus…
- Discontinuous or non-stationary sounds like fine or coarse crackles.

Now, we are going to detail the characteristics of the two more studied noises: wheezes and crackles [28].

### Characteristics of the respiratory cycles

Thanks to the description of analysis methods, Bahoura [3] also proposes his own definition of inspiration and expiration sounds’ characteristics: the frequency of tracheal sounds is located between 60 and 600 Hz for inspiration and between 60 and 700 Hz for expiration. Then, he proposes a Fourier transform with 4096 points and two types of representation of respiratory sound: the waterfall method with a representation of the spectrum in three dimensions (amplitude, frequency, time), and the spectrogram method that was mentioned above in this article. These representations generally allow to have a good visualization of respiratory cycles.

### Characteristics of wheezes

**Figure 2.**
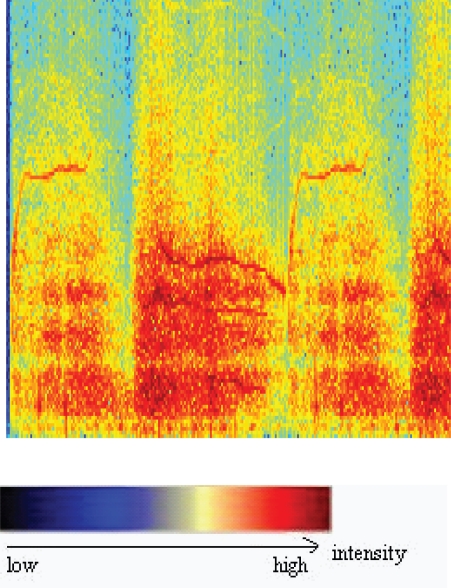
Spectrogram of a wheeze (bronchiolilies).

The identification of continuous adventitious breath sounds, such as wheeze in the respiratory cycle, is of great importance in the diagnosis of obstructive airways pathologies [29] (Fig. 2). In fact, Sovijarvi and coll. [1] indicate that wheezes can show acoustic characteristics symptomatic, not only of the presence of abnormalities in the respiratory system, but also of the severity and the location of the most frequently found airway obstructions in asthma and respiratory stenoses.

Wheezes, that Laennec calls dry wheezing groan, or wheezing, are sounds that have a duration (according to articles) greater than 50 ms [30] or 100 ms and lower than 250 ms [29].

The frequency of wheezes lies within 100 and 2500 Hz, with a fundamental frequency between 100 (or 400 [25]) and 1000 Hz [29] (or 1600 Hz [30]). On the other hand, [25] indicates that wheezes have a dominant frequency greater than 400 Hz, contrary to rhonchus whose dominant frequency lies within 200 Hz and below.

Finally, asthmatic subjects show wheezes during expiration phase; the latter have a duration range between 80 and 250 ms [17].

Fiz [31] and Albers [32] are able to identify objectively the presence of an obstructive pathology. Likewise, Meslier and Charbonneau [33] associate wheezes to the following pathologies:

- Infections such as croup (infection that generally affects infants from less than three years), whooping cough, laryngitis, acute tracheobronchilis
- Laryngo-, tracheo-, or bronchomalacia
- Laryngeal or tracheal tumours
- Tracheal stenosis
- Emotional laryngeal stenosis
- Foreign body aspiration
- Airway compression
- Asthma: wheeze detection in asthma [34], identification of nocturnal asthma [35],..

### Characteristics of crackles

Crackles correspond to short explosive sounds, generally associated with pulmonary disorders [36][37][38] (for instance lungs’ infection, pneumonia, pulmonary oedema…). They are generally generated during the airways opening that were abnormally closed during the inspiration phase, or during the closing in end-expiration.

Crackles detection is important in so far as their number is a possible indicator of the severity of a pulmonary affection [36], airways disorders [39]. Nevertheless, all the more as their number, their positioning in the respiratory cycle and the waveform of their signal are characteristics of the lung pathologic case [1].

**Figure 3.**
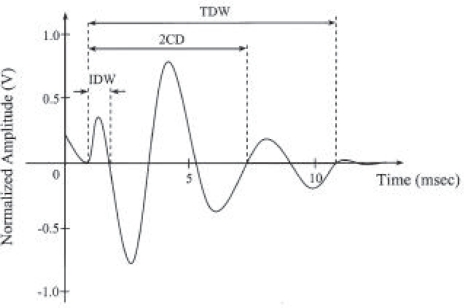
Waveform of a crackle.

Crackles generally begin with a width deflection, followed by a long and damped sinusoidal wave [40] [41] such as represented below (Fig. 3):

IDW or initial deflection width represents the duration between the beginning of the crackle and the first deflection.

2CD (two-cycle duration) is the duration from the beginning of the crackle to the date at which the waveform did two complete cycles.

TDW corresponds to the total duration of the signal crackle.

It is accepted ([25]) that the duration of a crackle is lower than 20 ms and the frequency range is between 100 and 200 Hz.

In addition, crackles can be divided into two families:

- **Fine crackles** (Laennec called them wet groan or “crépitations”) that are characterised, according to authors (respectively [42]and [43]) by IDW = 0,50 ms or 0,90 ms, 2CD = 3,3 ms or 6 ms, and TDW = 4 ms. They are exclusively inspiratory.
- **Coarse crackles** (“râle muqueux” or “gargouillement” according to Laennec) that are characterised by IDW = 1,0 ms, 2CD = 5,1 ms, TDW = 6,7 ms for [43] and by IDW = 1,25 ms, 2CD = 9,50 ms for [41]; they are generally inspiratory, but can also be expiratory.

Puerile and coll.’s article [36] describes the principal pathologies where crackles can be found:

- Pulmonary fibrosis (2CD <8 ms, frequency around 200Hz)
- Asbestosis (crackles’ duration around 10ms)
- Bronchiectasis (2CD >9 ms, they generally appear late in the inspiratory cycle and have a relatively long duration compared to the respiratory phase)
- COPD (2CD>9 ms, generally starting early in inspiration and ending before the mid-point of inspiration)
- Heart failure (2CD>10 ms)
- Pneumonia (2CD between 9 and 11 ms, they appear mid-point of inspiration)
- Sarcoidosis.

## Detection of Known Markers

Known markers are crackles and wheezes. The principal algorithm families of detection of these markers are summarised in Table 1.

Different analysis methods are described. We can quote temporal analysis of the waveform for crackles searching, and frequency analysis (Fourier transform, spectrogram in 2D or 3D [16], sonogram [48]) used for wheeze detection.

In techniques of spectral analysis, the main parameters are the average frequency of the spectrum, the frequency of maximal power, the number of dominant peaks, the factor of exponential decreasing. Finally, time-amplitude and time-frequency analysis are classically implemented thanks to a wavelet transform.

Among the complex solution, we can quote the use of a multi-layer perception in a neuronal network, genetic algorithms and a hybrid solution between both. The search of the parameters is performed through a learning method.

Guler and coll. [46] notice that the hybrid solution is the most effective.

Finally, Murphy and coll. [49] demonstrate that a multi-channel analyser (several sensors used simultaneously) is able to detect significant differences between the pulmonary sounds of patients suffering from pneumonia and patients without symptoms.

### Wheeze detection

As we explained before, reference [3] describes a spectral analysis technique for wheeze detection. In fact, the main characteristic of sounds stands in peaks of energy that can be visualized in the spectrum. The limits of this method stand in the existence, in normal pulmonary sounds, of peaks similar to those charactering wheezes. Consequently, an important rate of erroneous detections of generated.

The difficulties found during the automatic wheeze detection tools can be overcome thanks to a joint time-frequency analysis. As follows, the principle is: the detection in frequency domain of a peak that could correspond to a wheeze, will be followed by a second test in time domain in order to confirm true wheezes and reject erroneous ones.

According to Homs-Cobrera and coll. [50] significant parameters are frequencies and mean number of wheeze detected. They use parameters: number of wheezes, mean wheeze frequency with highest power peak, mean wheeze frequency with highest mean power, mean frequency, percentile of manoeuvre occupied by wheezes. The parameters are defined after dividing the frequency range into bands of 50 Hz from 150 to 200 Hz. Moreover, the present algorithm indicates that there is a significant correlation between the number of wheezes detected and the signal amplitude due to a simultaneous dependence between normalisation factor and fuzzy rules thresholds. Spectrograms provide a graphical time-frequency representation of the wheezes’ location. Nevertheless, this is not sufficient to objectively characterize sounds.

Another process of automatic wheeze detection was proposed [3][51]; it is based on wavelet packets decomposition, in two stages. First, it consists in frequency detection with wheeze extraction. Then, an inverse transform and a reconstruction of the useful signal; a time detection, here also makes it possible to eliminate false detection, generated by a superposition of spectral domains of some normal sounds and wheezes.

From spectrograms generated with recorded sounds, Lin and coll. [52] made a 2D bilateral filtering for edge-preserving smoothing. The results indicated a high efficiency of the system; authors ambition using this system for asthmatic patient monitoring and the study of airways’ physiology.

Similarly, a method of continuous wavelet transform is described in[29], combined with a scale-dependent threshold. This method seems to provide a higher good detection rate.

Meslier and Charbonneau’s article [33] also describes an automatic wheeze analysis and quantification of a spectral analysis. These algorithms are based on the definition of a threshold upon which the presence of peaks in frequency domain is characteristic of a wheeze. This threshold differs from one article to the other (thus, a peak can be characterised by a power 15 time greater than current average, or 3 times greater than average value. All these studies define constant threshold, based on power measurements.

Reference [53] confirms that frequency analysis alone generates a relative important number of erroneous detection. This article describes a new algorithm based on **auditory modelling**, called « frequency and duration dependent threshold (fddt) algorithm ». Parameters for average frequency and wheeze duration are obtained automatically. The notion of threshold depends on the frequency and duration introduced in a new wheeze detection algorithm. The threshold is no more based on global power, but on power corresponding to a particular frequency range.

The choice of energy instead of power was done according to previous studies results. Actually, the latter indicates that energies threshold was more suited to short-time sounds detection (lower than 200 ms).

### Crackles detection

Methods to detect crackles can be split into three major stages:

- a noise reduction filter is applied in order to the delete the residual stationary noise in a nonstationary signal,
- a search of the waveform corresponding to a crackle,
- detected crackles are classified in two categories: fine and coarse crackles.

Kayha and Yilmaz [63] propose an automatic system of crackles detection and classification. The proposed system uses a stationary/non-stationary filter and a wavelet packet transform (also called WPST-NST) that allows to isolate crackles from vesicular sounds.

Kawamura and coll.’s article [55] shows the existence of a correlation between respiratory sounds and high-resolution computed tomography findings. Two parameters, two cycles and the initial deflexion width of crackles were induced by time-expanded waveform analysis.

Kayha and coll. [56] describe a system based on increasing transient by an adaptative filter, and implementing nonlinear operators to wavelet decomposed lungs sounds.

Yeginerand and coll. also describe in their article [40] the utilisation of wavelet networks in order to model pulmonary crackles.

The algorithm proposed by [41] uses a stationary/non-stationary fuzzy-based filter (FST-NST). Results of the separation have a relatively good accuracy. The proposed algorithm deals with nonstationary crackles and fuzzy rules. The FST-NST filter was applied to sounds coming from three databases. First, crackles were separated from vesicular sound. Next, 27 “fuzzy if-then rules” were used. The results of the separation are reliable, objective, and high quality, in so far as the FST-NST filter automatically identifies the location of crackles in the original signal.

The reference [43] detects crackles and bowel sounds thanks to a fractal dimension analysis of the records. Results seem to be conclusive, and, moreover, robust to noise stress.

The comparison of the results coming from different methods is summarised in the Table 2.

The best results of classification were obtained using wavelet analysis.

The representation of Prony’s parameters indicates a correlation between the type of pathology, crackles occurrence compared to pulmonary volume, and Prony’s frequency [63].

In [59], the authors make a comparison between k-NN and ANN (artificial neural networks). They use different features extracted from the respiratory signal; actually each cycle is divided into six segments with three features: autoregressive coefficient, wavelet coefficient and crackles’ parameters.

Moreover, the performance of the classifiers was measured thanks to the following statistical parameters:

- sensitivity: number of pathological subjects classified correctly/total number of pathological subjects
- specificity: number of healthy subjects classified correctly/total number of healthy subjects
- accuracy: number of subjects correctly classified/total number of subjects.

### Respiratory cycle detection

In order to provide exploitable results, information must always be brought to a respiratory cycle [23]. Therefore, it is interesting to automatically detect inspiration/expiration phases. In [24], another characteristic of pulmonary signals is used : spectral power of pulmonary sounds during inspiration phase is higher than those during expiratory phase. This characteristic can be used, alone, to allow phase detection. Likewise, Chuah and Moussavi [4] use a processing of the average value of the spectral power to qualify respiratory cycle. This analysis is completed by the processing of the average value of tracheal spectral power to determine the beginning of respiration.

Moussari and coll. [64] use the average power spectrum of breath signal and the difference between average tracheal power spectrum and chest signal to detect respiratory phase. The results are between 31 and 69% good classification. Besides, the average power spectra difference between inspiration and expiration, in frequency range 150–450 Hz is maximum 10 dB. This method works fine for artificial sounds; nevertheless, it doesn’t allow to classify real auscultation sounds. Finally, in [65] they propose to qualify sound while using a fractal dimension and a parameter called “variance fractal dimension”.

Contrary to crackles or wheezes detection, the main methods of respiratory phase detection use artificial intelligence algorithms.

Thus, Guler and coll. [66] use a six-phase classification: begin, middle, end inspiration, and begin, middle, end expiration; this method lean on the utilisation of a multistage classification. The extracted features are autoregressive parameters and cepstral coefficients.

The development of such tool faces with two major difficulties:

- Respiratory signals are not stationary in so far as the volume of lungs is changing,
- Respiratory sounds present a great variability depending on age, mass, pathology evaluation state.

In [67], Guler and coll.’s base their study on a multilayer perceptron. On individual segment, it provides approximately 60% good recognition in expert phase.

In [68], Sa and Verbandt use two artificial independent neural networks (ANN): their algorithm is based in two neural networks ANNinspiration and ANNexpiration. First, a pre-processing is done; it normalises the signal in amplitude (between 0 and 1).

The next stage deals with the ANN with one hidden layer. The parameters are obtained thanks to a learning algorithm using back-propagation technics. Afterwards, a stage of post-processing is applied; it consists in removing the uncertain “1” that are situated between at least five “0” and inverly.

## Sound Classification

In lung medicine there is no universal pattern or parameters’ threshold indicating the presence or absence of a pathology. Therefore, Zheng and coll. [69] propose to establish a personalized pattern, combining information coming from sounds and other measurement applied to the patient. They aimed at recognizing pattern of pulmonary sounds. The method applied can be divided into two stages: characterize the variables that can be extracted from the waveform of pulmonary sound, and the changing in these variables that will provide information concerning the pattern variations.

Guler and coll. [46] focus on artificial intelligence technics; they combined neural network and genetic algorithm for analysis of lung sounds. First, they selected complete respiratory cycles, on which a PSD (Power Spectrum Density) of 256 was applied. Then, a multilayer perceptron (MLP) neural network was employed in order to detect the presence or absence of adventitious sounds (wheezes and crackles). The search of optimal parameters was done thanks to a learning method. Each sound is associated to several characteristics and to a diagnosis. 129 specific characteristics were checked of (PSD0,…, PSD128). Afterwards, different learning rules were used in order to associate characteristics and diagnosis.

In [59], Kahya and coll. make a comparison between k-NN *(k*-nearest neighbour) and ANN (artificial neural networks). They use different features extracted from the respiratory signal; actually each cycle is divided into six segments with three features: autoregressive coefficient, wavelet coefficient and crackles’ parameters.

Moreover, the performance of the classifiers was measured thanks to the following statistical parameters:

- sensitivity: number of pathological subjects classified correctly/total number of pathological subjects
- specificity: number of healthy subjects classified correctly/total number of healthy subjects
- accuracy: number of subjects correctly classified/total number of subjects.

Then, in [70], they added crackle parameters to the observed features in order to increase the performance of classification. As previousily K-NN and multinomiaux classifiers were used. It was observed that addition of crackles parameters to feature vectors and fusion of phase decisions improved classification results.

The study described in [71] focuses on four pathologies: asthma, bronchiectasis, COPD and pneumonia. The sound is divided into six sub-phases: early (30%), mid (40%), late (30%) inspiration and expiration. Classification experiments are applied to each sub-phase. Neural classifiers (multi-layer perceptrons MLP with hidden layer with ten nodes) were used with the following parameters: autoregressive parameters, error prediction, ratios of expiration/inspiration duration. The weigh and biases of the MLP are updated thanks to Levenberg-Marquardt’s optimization algorithm, that is one of the fastest. Then, the classification is realized in three stages: healthy/pathological classification, restrictive/obstructive classification, and classification between the pathologies (e.g. asthma and bronchiectasis). The accuracy is calculated by « global number of segment correctly classified/global number of segments ». Finally, the performance of classification are around 70 / 80%.

The study [72] aims at describing a preprocessing method to reduces the entry pattern size in neural networks, and to increase the performance of estimation or classification. The results indicate that wavelet expansions are significant signal sensors and allow to extract important features.

Pasika and coll. [73] realize classification of normal and adventitious sounds in two stages: linear prediction of coefficients, and features of the energetic envelope. Seven types of respiratory sound were thus classified, among which four normal sounds: vesicular breath sounds (V), bronchial breath sounds (B), broncho-vesicular breath sounds (BV), and tracheal breath sounds (T). The features extracted were: FFT, PDS estimation by means of linear prediction (LCP). Nevertheless, in this study, a manual decision of the inspiration/expiration periods was realized. The main objectives are: characterize quantitatively several respiratory sounds and provide an automatic classification method of these type of sounds. Finally, the diagnostic will be done by a physician, and based on the sound analysis associated with other diagnostic values. And on 105 experiments, only 5 generated errors.

Sezgin and coll. [74] use wavelet transform. The best samples are selected by dynamic programming. Then a Grow and Learn neural network is used for classification. The process of decision is made up of three stages: process normalization, feature extraction, artificial neural network by classification.

Actually, multi-layer perceptron is frequently used in biomedical signal processing. Nevertheless, they present three main drawbacks:

- backpropagation algorithm takes too long time during learning phase,
- the number of nodes in the hidden layers must be defined before the learning phase. The structure is not automatically determined by the training algorithm
- back-propagation algorithm may be caught by local minima, which decreases network performances.

## Factors Influencing Measurement

Several factors disturb the auscultation signal analysis [3]; they modify results and make comparison between research centers more difficult [75]:

- Age and corpulence of the patient
- Volume air changing in the lungs
- Location of sound capturing
- Breathing flow
- Position of the patient
- Characteristics of the measurement equipment.

### Age and corpulence

Differences due to age are all the more visible for infants. Elphinck and coll. [48] notice that stethoscope evaluation is not very accurate for wheeze and crackle detection [23]. Actually, audible respiratory sounds in early childhood have acoustics characteristics distinct from those generally heard in adults.

Therefore, Mazic and coll. [17] propose to use more objective methods to automatic detect wheeze in asthmatics infants, during forced breathing.

### Non stationary signals linked to lungs’ air volume variations

The static characterisation of the process evolves in respiratory cycle [34][76]. In fact, respiratory sounds are non-stationary in particularly because of the changing lung volume [67]. Thus, in order to correctly interpret the results, it is recommended to bring back to pulmonary air volume.

### Standardization of the measurement protocol

In order to overcome these limitations, it is proposed to define a semiology adapted to collect and analyse respiratory sounds. These works ended to a proposition of standardization that was proposed in a European project CORSA [33]. CORSA project describes auscultations’ points, type of sensors, filtering, sampling frequency, technique of FFT, definition of a spectrogram average, and used of standard flows.

## Figures and Tables

**Table 1.** The principal algorithm families of detection of the known markers.

**Signal**	**Characteristics and processing [7]**	**Analysis**
**Normal sounds**		
Lungs	Low-pass filtering (between 100 and 1000 Hz)	Periodogram (power spectral density—PSD), auto- regressive models [44]
Trachea	Noise with resonances [100, 3000 Hz]	
**Adventitious sounds**		
Wheezes	Sinusoid (range −100 and 1000Hz; duration >80ms)	Periodogram (PSD), STFT(short-time Fourier transform) [44], FFT, linear prediction of coefficients [45], genetic algorithms [46], neural networks [46], wavelet [29]
Ronchus	Series of sinusoid (<300 Hz and a duration >100 ms)	
Crackles	Wave deflection (duration typically <20 ms)	Temporal analysis [44], FFT, linear prediction of coefficients [45], fuzzy non stationary filter [45], genetic algorithms [46], neural networks [46], wavelet [43][47]
Snores		Temporal analysis, Periodogram (PSD) [44]
Stridors		Periodogram (PSD), STFT, auto regressive models [44]

**Table 2.** Methods developed to pulmonary sounds analysis.

**Methodology**	**Parameters**	**References**
Time-frequency analysis	Gaussien band width, peak frequency, total deflection width, maximal deflection width	[43] (correct classification level: 87,78%)
Time-frequency analysis	Gaussien band width, peak frequency, maximal deflection width	[43] (correct classification level: 90,5%)
Prony modeling	Parameters of the Prony model	[43][57] (correct classification level: 63,89%)
	Autoregressive coefficients	[58][59]
Wavelet transform	Wavelet scale	[43][47] (correct classification level: 93,9%)
	Wavelet transform fractal dimension based	[60]
	Wavelet transform stationary – non stationary	[61]
Fuzzy rule-based system – FST-NST	27 fuzzy rules	[42]
Artificial neural networks	Autoregressive coefficients, wavelet coefficients, crackles’ parameters	[59]
Empirical mode decomposition	Instrinsic mode function : local zero mean oscillating waves obtained by sifting process	[62]

## Conclusion/Future work

Today we are testing and studying different algorithm in the context of the ASAP project.

The next stage will consist in exploiting all the richness of the sound. This augmentation of the spectrum studied and linked to signal analysis techniques will allow the definition of new characteristic markers.

Previous studies demonstrate the need of performing an exhaustive scientific approach, that account of both the definition of a semiology, the consolidation of definition of known characteristics markers, the definition of common or even universal semantics, the development of determinist tools that will allow the detection of these markers. It is precisely the context of an ambitious study of in the so-called ASAP project (ANR n° 2006 TLOG 21 04 convention). This study is handled by a multidisciplinary teams including medical from CHRU of Strasbourg, IRCAD for web-based teaching tools, Alcatel-Lucent research teams for the development of the tools and algorithms, Laennext. Among the most identified outcome from the project, it is force in to create auscultation school hosted by the “Faculté de Médecine de Strasbourg”.
